# Extremely Low-Frequency Magnetic Fields Exposure Measurement during Lessons in Elementary Schools

**DOI:** 10.3390/ijerph17155284

**Published:** 2020-07-22

**Authors:** JinKyung Park, EunHye Jeong, GyeongAe Seomun

**Affiliations:** College of Nursing, Korea University, Seoul 02841, Korea; carpe@korea.ac.kr (J.P.); preaquaty@korea.ac.kr (E.J.)

**Keywords:** school, extremely low-frequency magnetic fields, measurement, exposure level

## Abstract

Schools are an important place for children’s exposure to electromagnetic fields, which may cause adverse health effects. To better understand environmental extremely low-frequency magnetic fields (ELF-MFs) exposure among elementary school students, we measured numeric values of ELF-MFs in five classrooms at four schools during digital learning class hours. The measurement of ELF-MFs was taken with an EMDEX II field analyzer. Specifically, we examined the level of exposure to ELF-MFs for each student’s seating position in the classroom. The results showed that ELF-MFs exposure levels were lower than those in the International Commission on Non-Ionizing Radiation Protection guidelines; however, there were significant differences in the level of magnetic field exposure at each school and at each student’s seat. The exposure to ELF-MFs at students’ seat positions was mostly caused by electrical appliances, electronic wiring, and distribution boxes, but the exposure level decreased as the distance increased. Therefore, it is important to design safe and appropriate environments for digital learning in schools, such as proper seating arrangements, to avoid ELF-MFs exposure to students as much as possible. Future studies should measure ELF-MFs levels in other areas and investigate the effects of exposure to ELF-MFs during school hours on children’s health.

## 1. Introduction

Electromagnetic fields (EMFs) exist wherever electricity is generated, transmitted or distributed in power lines or cables, or used in electrical appliances. Since the use of electricity is an integral part of modern life, these fields are ubiquitous in our environment [1]. The use of electrical devices has increased every year due to increasing demands and population growth; thus, the possibility of exposure to EMFs has also increased. Therefore, there is growing public concern that EMFs exposure may cause adverse health effects in children. Since the nervous and immune systems of children are still developing, it is possible that they could be more sensitive to EMFs [2,3,4,5].

Exposure to external electric and magnetic fields at extremely low frequencies induces electric fields and currents inside the body [1]. Several epidemiological studies have demonstrated an association between exposure to extremely low-frequency magnetic fields (ELF-MFs) and childhood leukemia [6,7,8,9,10,11], which has led to their classification by the International Agency for Research on Cancer (IARC) as a “possible human carcinogen” (Group 2B) [1,12] in 2002. The association between exposure to ELF-MFs and the risk of childhood cancer has been extensively investigated in epidemiological studies [13,14,15,16]. Studies on disorders of cognitive function [17,18,19], disorders of memory performance, headaches, and sleep disorders [20,21] in relation to EMFs have also been conducted.

It is essential that exposure limits be implemented in order to protect against the established adverse effects of exposure to ELF-MFs [1]. So far, only acute effects have been established, and there are several international exposure limit guidelines designed to protect against these effects [22,23]. In some countries, such as Switzerland, Germany, and Sweden, environmental protection facilities such as kindergartens and elementary schools have provisions for separation distances and facility limits to protect children from ELF-MFs emitted from power facilities, base stations, broadcasting stations, etc.

A school is an important place for children’s exposure to ELF-MFs because they spend 7–8 h a day there. Although ELF-MFs levels measured in schools are commonly lower than measured residential fields [24], in order to effectively assess health risks, such as the assessment of ELF-MFs, exposure in schools should not be neglected. Additionally, few studies have measured ELF-MFs at each student seat and confirmed the distribution in a classroom. Therefore, the purpose of this study was to measure the levels of exposure to ELF-MFs during class hours in order to investigate the digital learning environment at school. The study findings could be used as a basis for establishing a safe and appropriate environment in terms of ELF-MFs for children.

## 2. Materials and Methods

### 2.1. Research Design and Locations

This cross-sectional study was conducted to examine the distribution and level of exposure to ELF-MFs during digital learning class hours in elementary schools in Seoul, South Korea.

### 2.2. Research Objects

This study was conducted at elementary schools that indicated their intention to participate in the research. In December 2019, ELF-MFs were measured at five digital learning classrooms across four elementary schools. In each classroom, ELF-MFs were measured while maintaining the same environment as usual, and environmental factors such as temperature, arrangement and size of desks, and location of windows, doors, and electrical appliances were measured.

**Figure 1:**
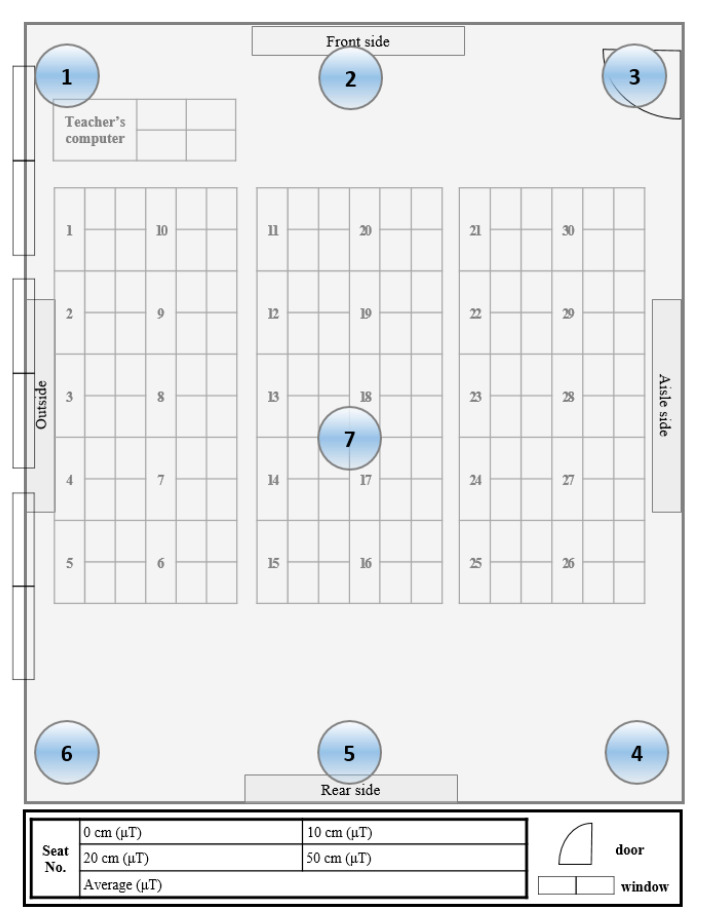
Measurement positions of extremely low-frequency magnetic fields in the classroom.

Additionally, the distribution of ELF-MFs in the classroom was assessed according to height (50 cm, 100 cm, 150 cm) at seven points (Figure 1). Additionally, ELF-MFs were measured at each student seat and the teacher seat for three separation distances (0 cm, 10 cm, 20 cm, 50 cm) from the computer monitor. ELF-MFs were also measured for electrical appliances and power supplies capable of emitting ELF-MFs in the classroom at each separation distance.

### 2.3. Extremely Low-Frequency Magnetic Fields Measurement

In this study, the professor and researchers at the Department of Health and Safety Engineering, experts in measuring EMFs, directly measured ELF-MFs. The equipment used for measuring ELF-MFs was EMDEX II (ENERTECH Inc., Greenville, IL, USA), which is typically used for measuring 60 Hz ELF-MFs such as those in household appliances. This instrument has an analytical sensitivity of 0.01 μT for the magnetic field at the maximum analysis sensitivity, and the maximum analytical value is 300 μT. The measurable frequency band is 40–800 Hz, which accounts for most of the electromagnetic emission in the very low frequency range, and the fixed frequency used in Korea is 60 Hz. The accuracy of the measured values is measured as ±3% magnetic field, respectively.

Determining the measurement conditions can be done using the Event marker button and Toggle button, and the measured conditions can be viewed through the liquid-crystal display (LCD) window. After the measurements were taken, data were transferred to the main computer, and the measurements were analyzed with EMCALC 2007 (Enertech Consultants, Campbell, CA, USA), an exclusive program for these data.

### 2.4. Statistical Analysis

The results of exposure to ELF-MFs were analyzed based on the electromagnetic exposure levels in the International Commission on Non-Ionizing Radiation Protection (ICNIRP) guidelines [12]. Descriptive statistics (i.e., maximum, minimum, median, mean, and standard deviation values) were used for describing the level of ELF-MFs exposure during class.

### 2.5. Ethical Considerations

This study was approved by the Institutional Review Board of Korea University (KUIRB-2019-0225-01).

## 3. Results

The results of measurements of exposure to ELF-MFs at seven points in each classroom during class are shown in Table 1. All of the measurements in all classrooms were lower than the ICNIRP guidelines. The maximum value of ELF-MFs exposure at the measurement point in the classroom was 0.28 μT at School 1 (0.14 % of the ICNIRP reference level, with a dominant measurement frequency of 60 Hz), and the maximum average exposure value in the classroom was 0.091 ± 0.025 μT in School 3. It was found that electronic devices and an electric distribution box emitting ELF-MFs were located at the point of highest ELF-MFs exposure level inside the classroom. When comparing ELF-MFs exposure for each school, the difference between the schools with the highest and lowest value was 0.085 μT.

**Figure 2:**
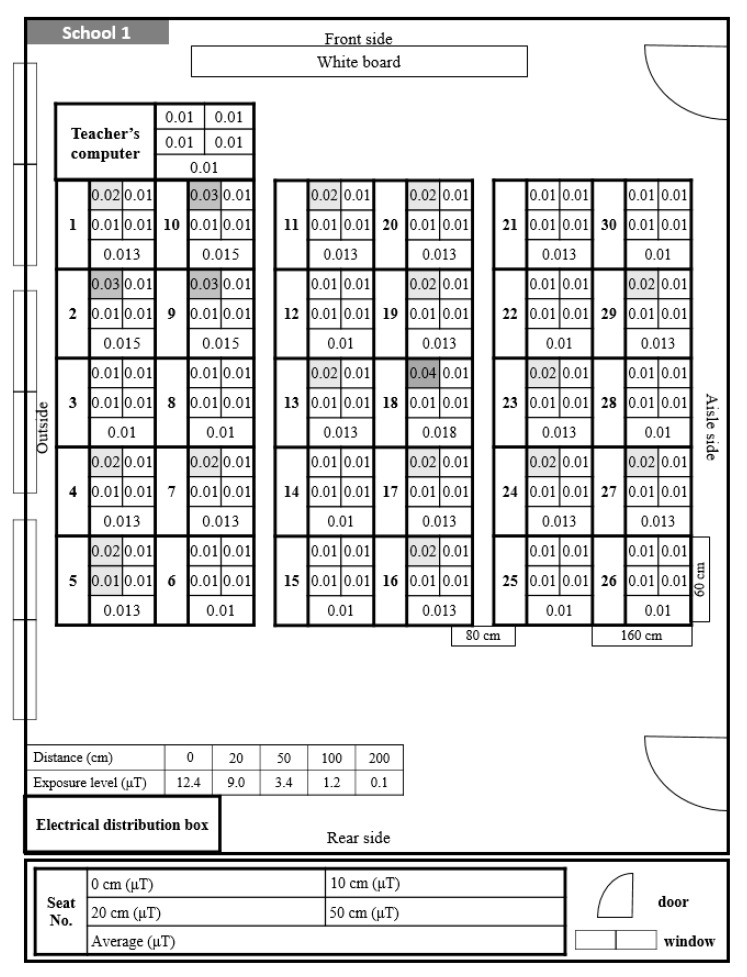
Distribution and exposure level of extremely low-frequency magnetic fields at each student seat in School 1. 0.01 is the lowest readout level, indicating <0.01. The darkening of the space indicates that the level of ELF-MFs exposure increase by 0.01.

The distribution of ELF-MFs exposure per student seat in the classroom in School 1 is shown in Figure 2. ELF-MFs were measured based on the distance from the monitor at all student seats and the teacher seat. At the teacher seat, there were several electronic devices, such as a large monitor and printer, and the level of exposure to ELF-MFs was higher than that at other seats. The level of electromagnetic exposure at the student seats near the teacher seat was also higher, and the exposure level of nearby students also appeared high. Additionally, the exposure to ELF-MFs was higher at the student seats near electronics and electrical wiring than at other seats.

When measuring the exposure to ELF-MFs in School 1 (Figure 2), the exposure level of ELF-MFs at the student seats near the teacher seat was high. However, when observing the posture of the students during the class, the distance from the monitor was about 20–50 cm; therefore, the exposure at that location was low at about 0.01 μT for all student seats. There was an electrical distribution box on the left back wall, showing an exposure to ELF-MFs of 1.24 μT. However, this did not affect the student seats because a sufficient distance was maintained.

**Figure 3:**
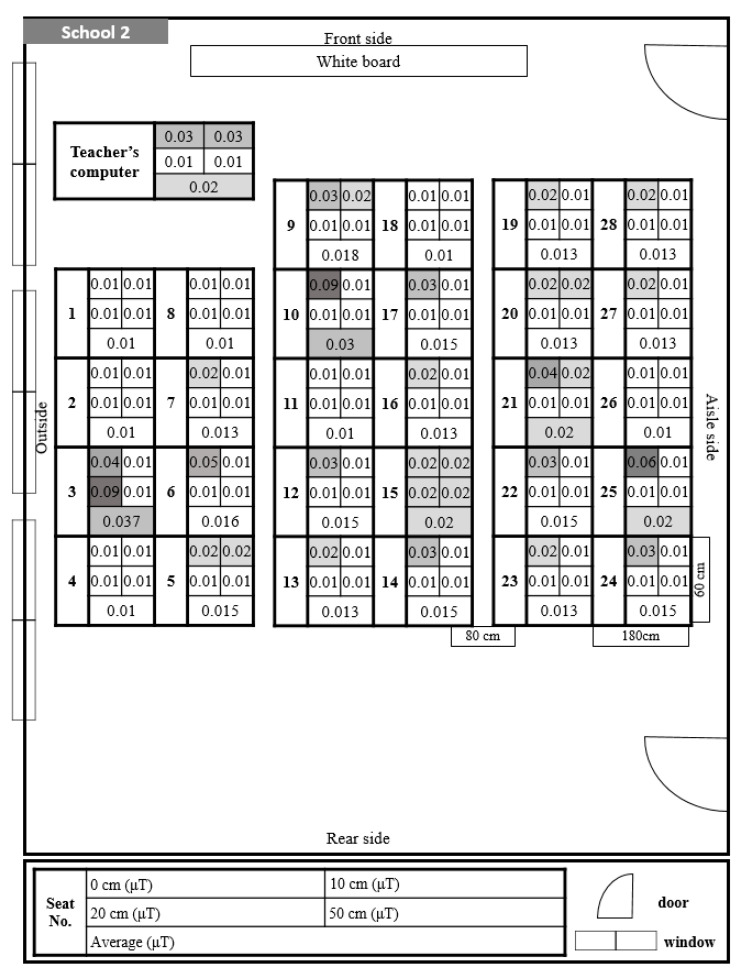
Distribution and exposure level of extremely low-frequency magnetic fields at each student seat in School 2. 0.01 is the lowest readout level, indicating <0.01. The darkening of the space indicates that the level of ELF-MFs exposure increase by 0.01.

In School 2, the level of exposure to ELF-MFs was found to be somewhat higher at the student seats in the middle of the classroom (Figure 3). However, all measurements of ELF-MFs at a distance of 20–50 cm were 0.01 μT.

**Figure 4:**
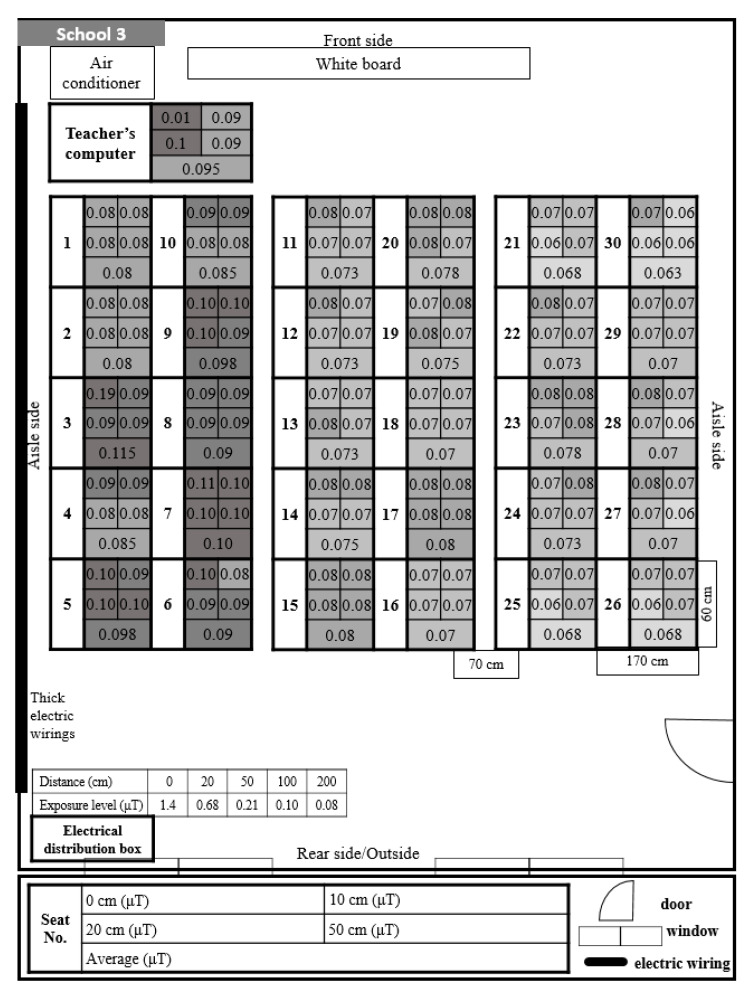
Distribution and exposure level of extremely low-frequency magnetic fields at each student seat in School 3. 0.01 is the lowest readout level, indicating <0.01. The darkening of the space indicates that the level of ELF-MFs exposure increase by 0.01.

School 3 showed the highest exposure level of ELF-MFs (Figure 4). The level was higher than 0.7 μT at all seats of students. Additionally, there was a higher exposure level on the left side of the classroom, which lowered gradually toward the right side. In this classroom, thick electric wirings passed along the left wall, and the floor had a floating structure under which the wiring was distributed.

**Figure 5:**
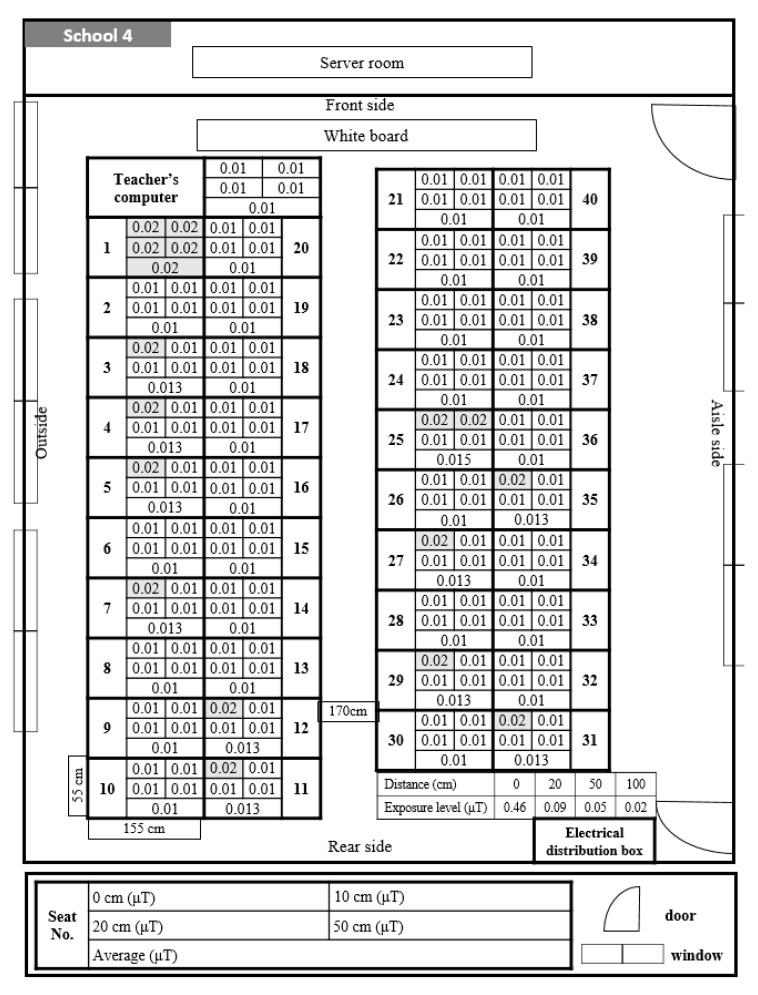
Distribution and exposure level of extremely low-frequency magnetic fields at each student seat in Classroom A at School 4. 0.01 is the lowest readout level, indicating <0.01. The darkening of the space indicates that the level of ELF-MFs exposure increase by 0.01.

**Figure 6:**
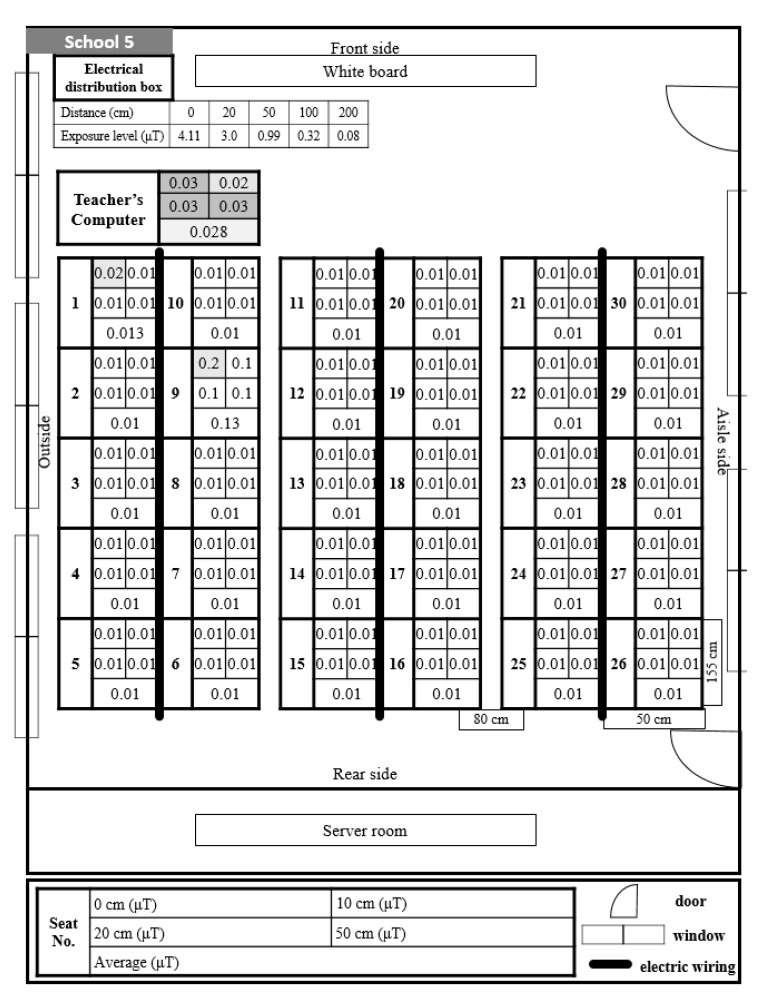
Distribution and exposure level of extremely low-frequency magnetic fields at each student seat in Classroom B at School 4. 0.01 is the lowest readout level, indicating <0.01. The darkening of the space indicates that the level of ELF-MFs exposure increase by 0.01.

Classroom A in School 4 was arranged with students facing each other and was used by the lower elementary school grades (Figure 5). All students’ seats showed low exposure levels of 0.01 μT, with the exception of one student seat in front of the teacher seat. In Classroom B in School 4, ELF-MFs exposure at the teacher seat was about 0.03 μT, and in all student seats, the exposure level was 0.01 μT (Figure 6). In this classroom, three electric wirings passed between rows of student seats from the front to the back; however, they were sealed with covers and did not expose the student seats to ELF-MFs.

## 4. Discussion

The average ELF-MFs exposure in all schools measured in this study was lower than the exposure standard level for the general public recommended by the ICNIRP guidelines [22]. These measurements were similar to the exposure levels in homes (0.025–0.07 μT in Europe and 0.055–0.11 μT in the USA) [1] and were higher than those recorded in the study of Tardón et al. (0.01 and 0.02 μT) [25] and Nassiri et al. (0.15 μT) [26]. However, in the present study, ELF-MFs exposure was measured in digital learning classrooms at seven points at different heights rather than at a single spot in the classroom, and a comparison with the results of previous studies is therefore difficult. This study proposes meaningful methods and criteria for measuring ELF-MFs exposure that can be used in future studies.

The exposure levels of ELF-MFs detected in this study could affect children’s perception [27] and is associated with a higher risk of childhood leukemia [13,14,15,16]. Disorders of cognitive function [17,18,19], disorders of memory performance, headaches, and sleep disorders [20,21] are also associated with these exposure levels. Although there has been no explicit evidence for adverse health effects in children by ELF-MFs exposure, since the nervous and immune systems of children could be more sensitive to ELF-MFs [2,28], children should avoid exposure to ELF-MFs as much as possible. Therefore, a reduction policy should be established to minimize exposure levels for children.

In this study, the distribution of ELF-MFs was measured at different distances from the student seat in the classroom where digital education using desktop personal computers was conducted. Since no previous study has measured the distribution of ELF-MFs in digital learning classrooms, these results could not be compared with other studies. However, in one study, the distribution of ELF-MFs was measured from a table personal computer in a general classroom, and the observed exposure level of ELF-MFs (from 0.01 to 0.02 μT) [29] was lower than that measured in the present study.

ELF-MFs arise most frequently from electrical wiring and electronic equipment, and the exposure level lowers as the distance increases [1]. Therefore, it is possible to know the level of exposure of students to actual ELF-MFs depending on where they are positioned and their distance from electrical wirings and electronic devices that can generate ELF-MFs in the classroom. Therefore, the level of exposure to ELF-MFs should be measured at the student seats in the classroom, and it should be determined whether the distance from the source of ELF-MFs affects exposure at the student seats.

In this study, exposure to ELF-MFs at the student seats was affected by the location of electronic devices, electrical wiring, and electrical distribution boxes, and the exposure was particularly high at the student seats near the teacher seat. In addition, when observing the posture of students during the class, since they were 20–50 cm away from the monitor, it was important to check the exposure level at that point.

In School 1, the exposure levels of ELF-MFs were 0.01 μT at a distance of 20–50 cm from the monitor. An exposure level of 1.24 μT was measured in the electrical distribution box at the left rear of the classroom; however, when the exposure level was measured according to the separation distance, it dropped to 0.01 μT at 2 m. Student seats were unaffected because they were more than 2 meters away. Therefore, it can be seen that even if there is electronic equipment that emits high ELF-MFs in the classroom, maintaining a sufficient separation distance can reduce the amount of exposure of students.

In School 2, the level of exposure to ELF-MFs was slightly higher in the student seats in the middle of the classroom located near the electrical wiring. However, based on the posture of students, the level of exposure to ELF-MFs at a distance of 20–50 cm from the monitor was measured as 0.01–0.02 μT. Additionally, the exposure level at the front and rear doors on the right was rather high (0.03 μT and 0.053 μT, respectively), but did not affect the student seats.

School 3 showed the highest exposure levels among all five classrooms, which was higher than 0.07 μT at every student seat. The maximum exposure level of ELF-MFs at the student seats was 0.115 μT. Moreover, the level was higher on the left side of the classroom and became lower toward the right side. There were thick unshielded electric wirings passing along the left wall and distributed under the floor. Therefore, the high exposure level of EMF-MFs could be caused by these electric wirings. The classrooms at Schools 2 and 4 also had electric wirings distributed under the floor; however, there was a low exposure level at all student seats. The high exposure level in School 3 may be due to the fact that the thick electric wirings were not properly shielded, and the student seats were not sufficiently separated from them. Therefore, it is necessary to maintain a sufficient distance from electric wirings to avoid the exposure to ELF-MFs, and it may be necessary to install proper shielding.

In Classroom A at School 4, exposure levels of 0.01–0.02 μT were measured at all student seats, which was the lowest average ELF-MFs exposure level. Unlike other classrooms, the monitors were placed facing each other, which has the effect of reducing students’ level of exposure to ELF-MFs from the back computer.

Classroom B at School 4 showed exposure levels of 0.01 μT at all student seats; however, there was an electric distribution box behind the teacher seat, leading to a high exposure level (0.09 μT). This had an effect on the level of exposure to ELF-MFs at the teacher seat but did not affect the student seats. Additionally, in this classroom, three electric wirings passed between the rows of student seats from the front to the back of the classroom; however, they were shielded with metal covers, and the student seats were not exposed to ELF-MFs.

The main sources of ELF-MFs in the classroom are electrical equipment (e.g., electric distribution boxes, electrical wirings) and electrical appliances (e.g., air conditioners, heaters, air purifiers, projector screens) [30,31]. As for the results of this study, the factors contributing to the strength of ELF-MFs in each school classroom were the number of electrical appliances used in the classroom and the separation distance from electrical appliances, electrical wiring, and electric distribution boxes. Our results indicate that exposure to ELF-MFs can be avoided if a sufficient distance is maintained, which can be identified by measuring exposure levels at each separation distance from the device emitting ELF-MFs. Additionally, it is important to measure exposure to EMF-MFs in the digital learning classroom and check the level of exposure at student seats to place them adequately.

Our results indicate that two classrooms showed higher exposure levels than other classrooms. However, it has been shown that the level of exposure to ELF-MFs can be lowered by maintaining a sufficient distance from electronic devices and electric distributors, and properly shielding and positioning electrical wirings. Therefore, it is important to manage the ELF-MFs in schools, including recommendations such as proper seating arrangement to avoid ELF-MFs exposure to students as much as possible in the classroom. Based on the results from this study, appropriate seating is suggested, as it may be helpful in creating a safe and appropriate learning environment in relation to ELF-MFs.

A limitation of this study was that the results cannot be generalized, as the exposure level of ELF-MFs was measured only in five digital learning classrooms. Therefore, it is necessary to measure exposure to ELF-MFs in various educational environments. Moreover, research needs to be conducted on the development of guidelines to manage exposure to ELF-MFs in schools and the effects of exposure to ELF-MFs during school hours on children’s health.

## 5. Conclusions

As digital learning increases, questions have been raised about the effects on children of exposure to ELF-MFs from electronic devices used in digital learning environments. Therefore, in order to understand the distribution of ELF-MFs intensity in the classroom, the exposure level of ELF-MFs was measured at each student seat in five classrooms and compared with the ICNIRP guidelines. All five classrooms in the study showed ELF-MFs exposure levels lower than the ICNIRP standard, but two classrooms showed higher exposure levels than other classrooms, with such high exposure levels being due to the effects of electric distribution boxes, electrical wirings, and electronic appliances. In the other three classrooms, these devices were also present; however, ELF-MFs exposure levels were low because of a sufficient distance and proper shielding of electrical wirings. Therefore, in order to minimize the level of children’s exposure to ELF-MFs, it is necessary to manage environments in school, such as measuring ELF-MFs, minimizing electronic devices and maintaining separation distance from them, and properly placing student seats.

## Figures and Tables

**Table 1:** Extremely low-frequency magnetic fields (ELF-MFs) exposure levels in the classrooms in each school.

Schools	Points	Level of Exposure to ELF-MFs (μT)			Mean of Level of Exposure to ELF-MFs		Remarks	No. of Seats	Temperature (°C)
		50 cm	100 cm	150 cm					
1	1	0.01	0.01	0.01	0.01	0.044 ± 0.066		30	24
	2	0.01	0.01	0.01	0.01				
	3	0.01	0.01	0.01	0.01				
	4	0.06	0.05	0.03	0.047		Electrical wirings		
	5	0.11	0.18	0.28	0.19		Electrical wirings		
	6	0.05	0.04	0.01	0.033		Electrical distribution box		
	7	0.01	0.01	0.01	0.01				
2	1	0.01	0.01	0.01	0.01	0.019 ± 0.017		28	23
	2	0.01	0.01	0.01	0.01				
	3	0.03	0.03	0.03	0.03		Entrance		
	4	0.01	0.04	0.11	0.053		Entrance		
	5	0.01	0.01	0.01	0.01				
	6	0.01	0.01	0.01	0.01				
	7	0.01	0.01	0.01	0.01				
3	1	0.09	0.15	0.16	0.133	0.091 ± 0.025	Air conditioner	30	25
	2	0.09	0.09	0.09	0.09				
	3	0.03	0.08	0.08	0.063				
	4	0.13	0.09	0.09	0.103		Electrical distribution box		
	5	0.03	0.08	0.08	0.063				
	6	0.11	0.10	0.10	0.103		Heater		
	7	0.08	0.08	0.08	0.08				
4A	1	0.01	0.01	0.01	0.01	0.01		40	25
	2	0.01	0.01	0.01	0.01				
	3	0.01	0.01	0.01	0.01				
	4	0.01	0.01	0.01	0.01				
	5	0.01	0.01	0.01	0.01				
	6	0.01	0.01	0.01	0.01				
	7	0.01	0.01	0.01	0.01				
4B	1	0.19	0.05	0.03	0.09	0.021 ± 0.034	Electrical distribution box	30	24
	2	0.01	0.01	0.01	0.01				
	3	0.01	0.01	0.01	0.01				
	4	0.01	0.01	0.01	0.01				
	5	0.01	0.01	0.01	0.01				
	6	0.01	0.01	0.01	0.01				
	7	0.01	0.01	0.01	0.01				

0.01 is the lowest readout level, indicating <0.01.; A is the classroom for lower grade students; B is the classroom for higher grade students.
